# Brain Correlates of Chinese Handwriting and Their Relation to Reading Development in Children: An fMRI Study

**DOI:** 10.3390/brainsci12121724

**Published:** 2022-12-16

**Authors:** Jun Zhang, Liying Kang, Junjun Li, Yizhen Li, Hongyan Bi, Yang Yang

**Affiliations:** 1 College of Education, Capital Normal University, Beijing 100048, China; 2 College of Preschool Education, Capital Normal University, Beijing 100048, China; 3 CAS Key Laboratory of Behavioral Science, Center for Brain Science and Learning Difficulties, Institute of Psychology, Chinese Academy of Sciences, Beijing 100101, China; 4 Department of Psychology, University of Chinese Academy of Sciences, Beijing 100049, China

**Keywords:** handwriting, reading, children, Chinese, fMRI

## Abstract

Handwriting plays an important role in written communication, reading, and academic success. However, little is known about the neural correlates of handwriting in children. Using functional magnetic resonance imaging (fMRI) and a copying task, we investigated regional brain activation and functional lateralization associated with Chinese handwriting in children (N = 36, 9–11 years old), as well as their relations to reading skills. We found significant activation of the bilateral frontal motor cortices, somatosensory cortex, intraparietal sulcus (IPS), fusiform gyrus (FuG), and cerebellum during handwriting, suggesting that an adult-like brain activation pattern emerges by middle childhood. Moreover, children showed left-lateralized and bilateral activation of motor regions and right-lateralized activation of the FuG and cerebellum during handwriting, suggesting that functional lateralization of handwriting is not fully established by this age. Finally, the activation of Exner’s area and the lateralization of the IPS and cerebellum during handwriting were correlated with reading skills, possibly representing a neural link between handwriting and reading in children. Collectively, this study reveals the brain correlates of handwriting and their relation to reading development in Chinese children, offering new insight into the development of handwriting and reading skills.

## 1. Introduction

Handwriting is a combination of linguistic and motor processes that provides essential support for written communication, reading, and academic achievements [1,2]. Moreover, a large body of research has revealed an association between handwriting impairments and a variety of neurological disorders, such as dyslexia [3], attention deficit hyperactivity disorder (ADHD) [4], and autism spectrum disorder (ASD) [5]. Thus, skilled handwriting is a critical developmental milestone that merits attention from researchers and educators. Furthermore, the importance of skilled handwriting in children is of particular concern in the digital age because it can be easily neglected due to the widespread use of digital devices. 

Previous functional imaging studies have revealed brain architecture that supports handwriting, showing that a distributed brain network is engaged in handwriting, including the left posterior middle frontal gyrus (also referred to as Exner’s area), primary motor cortex, postcentral gyrus, intraparietal sulcus (IPS), superior parietal lobule (SPL), usiform gyrus (FuG), and cerebellum [6,7]. Specifically, Exner’s area is regarded as a handwriting-specific brain region that transforms information from orthographic codes into graphic programs [8,9] and orthographic working memory [10]. In addition, the IPS/SPL and cerebellum are the neural substrates of motor execution involved in handwriting [6,7]. The FG is involved in writing and reading processes [11,12], and is thought to support orthographic access for handwriting [10,12]. In addition, a few studies have examined the functional lateralization of core brain regions associated with handwriting, showing that Exner’s area and the IPS/SPL exhibit left-lateralized activation during handwriting in right-handers [7,9], but right-lateralized activation in left-handers [13]. 

A few studies of children have examined the developmental trajectory of the brain system supporting handwriting. For example, a recent study directly compared brain activation during handwriting between French-speaking children and adults and found differences in activation in the left FG, right precentral gyrus, and right anterior cerebellum. Moreover, the children did not show lateralization of activation during handwriting [14]. Another study further examined the development of brain activation patterns associated with handwriting in young children (aged 5.5–8 years old). Children and adults exhibited differences in the activation of the left IPS and ventral precentral gyrus, suggesting that the dorsal neural network that supports handwriting exhibits a development trend [15]. These findings suggest that the changes in regional activation and functional lateralization of motor and visual regions are associated with handwriting development. 

Cognitively, handwriting is the reverse of reading; thus, the contributions of handwriting to reading development have attracted attention. Several lines of evidence suggest that handwriting plays a vital role in reading development [16,17]. For example, behavioral studies have shown that handwriting speed is positively associated with reading skills [16,18]. Moreover, handwriting training improves letter recognition to a greater extent than passive viewing of letters or typing [19]. These benefits may accrue through two avenues. First, handwriting helps to form motor memories, which may be reactivated to facilitate subsequent recognition of written words [16,20,21,22]. The motor skills of handwriting can help to distinguish visually similar letters (e.g., ‘b’ and ‘d’) based on the differences in the motor programs [23]. Second, visual processing of the elements of written stimuli is inherently involved in handwriting, which may elaborate orthographic representation for reading [16]. For example, handwritten outputs are highly variable; therefore, viewing many examples of handwritten letters can train the visual category of written letters, which ultimately facilitates visual recognition [20]. 

At the neural level, direct evidence of a writing-and-reading connection is derived from the activation of shared regions (the premotor cortex) by both handwriting and visual letter recognition [24,25,26]. In addition, the activation of the motor cortex is positively correlated with letter recognition [15]. Furthermore, a large body of evidence indicates that handwriting training increases brain activation in the motor (e.g., ventral and dorsal premotor cortex and IPS) and visual regions (e.g., the FG and lingual gyrus) involved in reading processing [23,27,28,29]. 

Chinese is a logographic writing system that differs from alphabetic languages in many aspects. In Chinese, the basic functional unit (i.e., grapheme) is the character. Characters are composed of radicals and strokes, yielding a visually complex square configuration. At the systematic level, Chinese lacks regular correspondence between phonology and orthography; thus, there are high numbers of homophones. These features may result in unique brain mechanisms that support Chinese handwriting. For example, a functional magnetic resonance imaging (fMRI) study showed that Chinese handwriting elicited greater activation of the left MFG than English handwriting [30]. Moreover, in addition to the left FuG being identified as involved in the handwriting of alphabetic languages [7], the right FuG is involved in Chinese handwriting [31,32]. 

Due to these characteristics of Chinese, a typical strategy to learn Chinese characters is repeated copying [33]. Thus, the relationship between handwriting and reading development is expected to more pronounced in Chinese than in alphabetic languages [16]. In line with this view, an fMRI study showed that the activation of the left middle frontal gyrus during handwriting was greater in Chinese than in English [30]. Likewise, another fMRI study reported that visual recognition of Chinese characters learned by handwriting involved greater activation of the bilateral sensorimotor cortex and right visual region than characters learned by writing in pinyin (alphabetic writing system) [29]. However, the above findings mostly come from studies of adults. The neural basis of handwriting in Chinese children and its relation to reading development remain largely unknown. 

The present study sought to answer two research questions. First, we aimed to identify brain correlates of handwriting in Chinese children. To this end, we measured brain activation and functional lateralization during a copying task, a task widely used to identify the brain substrates of handwriting [32,34]. Based on findings from alphabetic languages [14], we expected to observe the involvement of Exner’s area, the ventral motor cortex, IPS/SPL, FuG, and cerebellum during handwriting in children, similar to that in adults. In addition, we expected to find left-lateralized activation of Exner’s area and the IPS/SPL, bilateral activation of the FuG, and right-lateralized activation of the cerebellum. Second, we aimed to determine how the neural circuitry of handwriting relates to reading development by using a series of brain-behavior correlation analyses. Character recognition and reading speed, two representative indices of reading skills, were examined. 

We hypothesized that both regional activation and functional lateralization of motor (e.g., Exner’s area, the IPL/SPL, and cerebellum) and visual regions (e.g., the FuG) would be related to reading skills in children.

## 2. Methods

### 2.1. Participants

Thirty-six children (15 males and 21 females; age range = 9.15–11.11 years) were recruited from 5 elementary schools in Beijing, China, to participate in this study. All participants were native Chinese speakers. They were right-handed, as determined by the Edinburgh Handedness Inventory [35], and had normal or corrected-to-normal vision. All participants were typically developing children without a history of psychiatric or neurological disorders. Their nonverbal intelligence quotient (IQ) was assessed by the Combined Raven’s Progressive Matrices Test (CRT). The study was approved by the Ethics Committee of the Institute of Psychology, Chinese Academy of Sciences (No. H22011). Informed consent was obtained from each adult participant and from each child participant’s guardian prior to the experiment. Detailed information on the participants is presented in Table 1.

### 2.2. Handwriting Tests

A series of behavioral tests were conducted to assess the children’s reading and handwriting skills. All tests have been introduced in detail in a prior study [3]. Briefly, handwriting skill was assessed by copying tests and handwriting fluency tests. In the copying tests, participants were instructed to copy 48 Chinese characters that varied in frequency and visual complexity. Writing speed and quality were evaluated. Writing quality was assessed by 2 independent examiners on a 7-point Likert scale, based on 6 dimensions, including stroke form, slant, organization of radicals, neatness, average size, and overall appearance [32,36]. In the handwriting fluency tests, participants were instructed to write out digits in capitals with Chinese characters and to write the Chinese sentence “妈妈永远爱我” (“Mommy loves me forever”) as quickly and as legibly as possible within 1 min. The score was based on the number of digits or characters written correctly. 

### 2.3. Reading Skill Tests

Reading ability was evaluated using a character recognition test and a reading fluency test. Character recognition was assessed by using the Character Recognition Measures and Assessment Scale [37], a widely used test to assess Chinese reading [38,39]. In the reading fluency test, participants were instructed to read 160 characters aloud as quickly and accurately as possible within 1 min. Reading speed was calculated by the number of characters correctly read. 

### 2.4. fMRI Analysis

#### 2.4.1. Stimuli and Procedures

Participants performed a delayed copy task during the fMRI scan. Details regarding the task design are available in our prior study [3]. Thirty-two characters were selected for the task, half of which were high-frequency characters (HFCs), and half were low-frequency characters (LFCs). Motoric complexity, characterized by the number of strokes, was matched between HFCs and LFCs (HFCs: mean strokes (standard deviation, SD) = 5.56 (0.63); LFCs: mean strokes (SD) = 5.35 (0.72)). 

Each participant underwent 2 fMRI “runs”, with 2 blocks of handwriting and 2 blocks of symbol drawing, which were presented in a pseudo-random order. Each block consisted of an instruction presenting for 2 s and 4 subsequent trials. In each trial, a central fixation across was presented for 0.5 s, followed by the presentation of a character for 1.2 s. Then, a blank screen was displayed during a delay period of 0.5 s, followed by the central appearance of the cursor for handwriting or drawing at the beginning of a response period of 5.3 s. Interspersed between the 2 task blocks, 8 12-s blocks of a central fixation cross were included to collect baseline activity. 

Handwriting was recorded using a tablet system specifically developed for use in fMRI experiments [40]. Participants were instructed to write characters and draw symbols matched in duration and size, and to reduce the motion of their upper arm and forearm as much as possible. Visual feedback was provided on the display screen during writing and drawing tasks. 

#### 2.4.2. Imaging Data Acquisition

Functional and structural MRI data were acquired on a 3 T MRI system (MAGNETOM Prisma^fit^, Siemens, Erlangen, Germany) at the Beijing MRI Center for Brain Research of the Chinese Academy of Sciences. Functional MRI time series data with blood oxygenation level-dependent (BOLD) signals were acquired using a 2-D, T2*-weighted, gradient-echo echo planar imaging (EPI) sequence [41] using the following parameters: repetition time (TR) = 1000 ms, echo time (TE) = 30 ms, slice thickness = 2.2 mm, in-plane resolution = 2.2 mm × 2.2 mm, flip angle (θ) = 45°, and slice number = 64 axial slices. High spatial resolution anatomical images were acquired using a 3-D, T1-weighted, magnetization-prepared rapid acquisition gradient echo (MPRAGE) sequence with the following parameters: TR = 2200 ms, TE = 3.49 ms, slice thickness = 1 mm, inversion time (TI) = 1000 ms, in-plane resolution = 1.0 mm × 1.0 mm and (θ) = 8°.

#### 2.4.3. Preprocessing

Image preprocessing and statistical analyses were conducted using SPM12 freeware (http://www.fil.ion.ucl.ac.uk/spm/, 1 January 2022, Wellcome Department of Cognitive Neurology, University College London, London, UK). The fMRI time series data were corrected for head motion, spatially transformed into Montreal Neurological Institute (MNI) stereotactic space with cubic voxels at 2 mm × 2 mm × 2 mm spatial resolution and spatially smoothed using an isotropic Gaussian kernel template with 6 mm full-width at half-maximum. Excessive head motion (>3 mm translation or > 3° rotation) in both fMRI runs resulted in the exclusion of 6 participants from the data analysis. In addition, data from 7 child participants were excluded due to poor quality T1-weighted images. Thus, the statistical analysis included data from 23 children. 

### 2.5. Statistical Analysis

#### 2.5.1. Brain Activation Analysis

At the first level, the general linear model (GLM) method was used. The GLM design matrix including the block design time series convolved with a canonical hemodynamic response function was applied to generate activation maps for handwriting and drawing conditions. In addition, head movement parameters (6 parameters estimated during the motion correction step) were included in the design matrix as nuisance covariates to minimize residual motion artifacts. The data were high-pass filtered at 0.008 Hz. At the second level, brain activation maps for the comparisons between handwriting/drawing tasks and rest and between HFC and LFC conditions were acquired using a 1-sample *t* test. he voxelwise threshold was set as *p* < 0.05 after applying familywise error (FWE) correction for multiple comparisons and the cluster size was set to at least 50 activated voxels.

#### 2.5.2. Lateralization Analysis 

The lateralization index (LI) of brain activation was calculated using the LI toolbox [42]. This analysis was applied to the regions of interest (ROIs) related to handwriting processing, including 2 nodes in Exner’s area (MNI coordinates, anterior: −26, 2, 58; 26, 2, 58; posterior −22, −8, 54; 22, −8, 54), the posterior middle frontal gyrus (MFG) (−50, 6, 26; 50, 6, 26), the left IPS/SPL (−32, −38, 56; 32, −38, 56), the FuG (−46, −62, −12; 46, −62, −12), and the cerebellum (−18, −52, −22; 18, −52, −22). The LI was determined using a bootstrap method implemented with thousands of comparisons across thresholds between the 2 hemispheres and calculated using the equation: (Left − Right)/(Left + Right). The HFC and LFC conditions were combined into a single handwriting condition, because no difference was detected in regional activation between the HFC and LFC conditions (see the Section 3). According to previous studies [43,44], left lateralization was defined as LI > 0.2, right lateralization was defined as LI < −0.2, and bilateral activation was defined as −0.2 ≤ LI ≤ 0.2. At the group level, nonparametric tests were applied to examine the significance of lateralization and the differences in the lateralization degree between conditions for each ROI. The significance was defined as *p* < 0.05.

#### 2.5.3. Correlation between Brain Activation and Functional Lateralization during Handwriting and Reading 

To explore how the neural circuitry of handwriting relates to that of reading, we conducted a series of correlation analyses between regional activation and lateralization of key regions involved in handwriting and reading including Exner’s area, the MFG, the left IPS, the bilateral FuG, and the right cerebellum [7]. Spherical ROIs were created with a 6-mm radius. First, we examined the association between regional activation of these ROIs and reading skills. Contrast estimates and LI values were extracted from the contrast of handwriting/drawing versus rest from each ROI; these values were correlated with character recognition and reading speed, respectively, using partial correlation analyses after controlling for age and IQ. All statistical analyses were performed using SPSS 16.0. The threshold was set at *p* < 0.05 after applying a false discovery rate (FDR) correction for the 7 ROIs.

#### 2.5.4. Post Hoc Power Analysis

Furthermore, post hoc power analysis was conducted to determine the validity of lateralization based on α = 0.05 and the current sample size (N = 23) using G*Power (Version 3.1, http://www.gpower.hhu.de/, 1 January 2022) [45].

## 3. Results

### 3.1. Behavioral Results

The results of the reading and handwriting tests are presented in Table 1. Since the scores of some handwriting and reading skill tests did not exhibit a normal distribution as evaluated by Shapiro-Wilk tests (writing quality: *W* = 0.94, *p* = 0.057; copying speed: *W* = 0.83, *p* < 0.001; writing fluency (sentence): *W* = 0.97, *p* = 0.385; writing fluency (digits): *W* = 0.92, *p* = 0.012; character recognition: *W* = 0.97, *p* = 0.356; reading speed: *W* = 0.92, *p* = 0.012), the scores were first normalized using the Blom formula. Partial correlation analyses indicated that the handwriting fluency of digits was significantly correlated with reading speed (r = 0.36, *p* = 0.032) after controlling for age. Moreover, character copying time was marginally correlated with reading speed (r = −0.283, *p* = 0.099).

**Figure 1:**
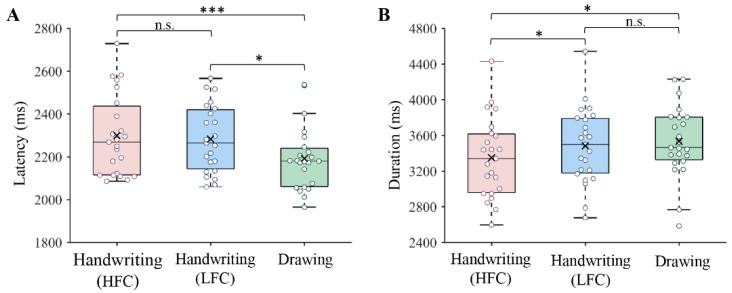
Writing latency (**A**) and duration (**B**) in different conditions during the fMRI scan. HFC = high-frequency character, LFC = low-frequency character and ms = millisecond. The ‘**×**’ in the boxplots represents the mean value. * *p* < 0.05, *** *p* < 0.001. n.s. = not significant.

The in-scanner task performance is presented in Figure 1. Writing latency and duration were analyzed. The former was defined as the time between the appearance of the response screen and the start of the response, and the latter was defined as the time from the start of the response to the end of the written response. Repeated-measures analysis of variance (ANOVA) showed that there were significant differences in writing latency (*F*(2, 44) = 7.33, *p* = 0.002) and writing duration (*F*(2, 44) = 4.44, *p* = 0.018) between conditions. Post hoc analysis showed that the writing latency for handwriting was longer than that for drawing symbols (HFCs: *p* < 0.001; LFCs: *p* = 0.017). However, there was no significant difference in writing latency between the HFC and LFC conditions (*p* = 0.580). Moreover, the writing duration of HFCs was shorter than that of LFCs (*p* = 0.013) and symbols (*p* = 0.020), while there was no difference in writing duration between writing LFCs and drawing symbols (*p* = 0.463).

### 3.2. Brain Activation Results

**Figure 2:**
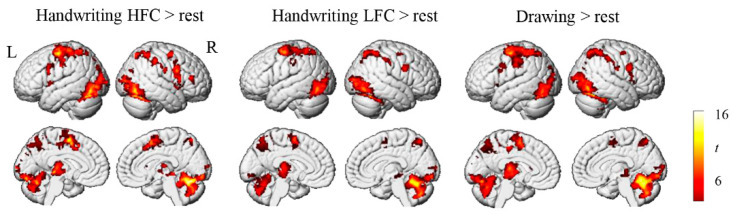
Brain activation during handwriting and drawing. HFC = high-frequency character, LFC = low-frequency character, L = left, R = right.

First, we explored the overall brain activation patterns associated with handwriting and drawing by contrasting activation patterns during the task conditions with those during rest. Children showed significant activation in the frontal motor cortex, somatosensory cortex, IPS/SPL, inferior occipital gyrus, FuG, and cerebellum when writing characters and drawing symbols (Figure 2). Next, we examined differences in brain activation between conditions (handwriting versus drawing and HFC versus LFC). We found no significant differences in regional activation between handwriting and drawing, or between the HFC and LFC conditions, even though a lenient statistical threshold of *p* < 0.001 (not corrected for multiple comparisons) was used. 

### 3.3. Functional Lateralization Results

**Figure 3:**
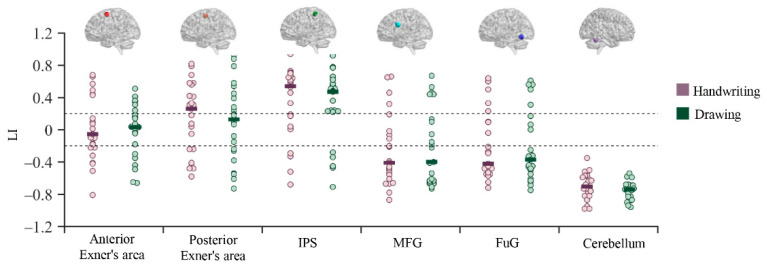
Activation lateralization values of each ROI during handwriting and drawing. Each dot represents one participant, and the short dark line represents the median value. The upper and lower dotted lines correspond to lateralization values of −0.2 and 0.2, respectively. IPS = inferior parietal sulcus, MFG = middle frontal gyrus, FuG = fusiform gyrus.

Since the LI values in most regions did not follow a normal distribution, we used the median (rather than the mean) to conduct comparisons. The median LI values in each ROI in the different conditions are presented in Figure 3. The anterior Exner’s area exhibited bilateral activation during handwriting and drawing. However, the posterior Exner’s area exhibited a trend of left-lateralized activation during handwriting, but not when drawing symbols. Similarly, the IPS showed left-lateralized activation during both handwriting and drawing. In contrast, the posterior MFG, FuG, and cerebellum exhibited right-lateralized activation during both handwriting and drawing. However, the Wilcoxon signed-rank test showed that only the LI value of the cerebellum was significantly different from −0.2 during handwriting (*W* = 0, *p* < 0.001, power > 0.99) and drawing (*W* = 0, *p* < 0.001, power > 0.99). Furthermore, between-condition comparisons revealed that there were no significant differences in activation lateralization in any of the ROIs between handwriting and drawing tasks, suggesting that hemispheric lateralization of handwriting is comparable between handwriting and drawing processes in children.

### 3.4. Association between Brain Correlates of Handwriting and Reading 

**Figure 4:**
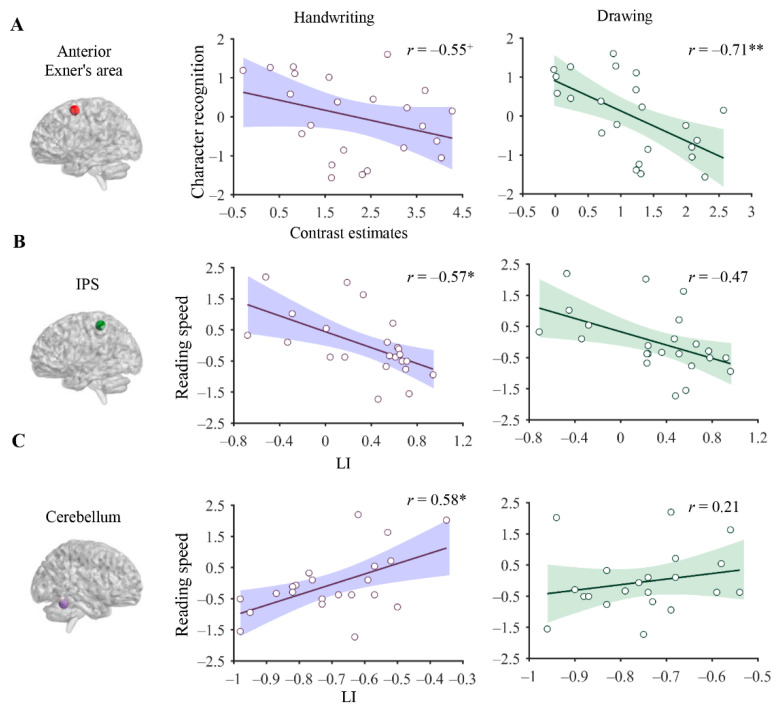
Scatter plots displaying the correlations of brain activity associated with handwriting/drawing and reading skills. Correlations of activation in the anterior Exner’s area during handwriting/drawing versus rest with character recognition scores (**A**). The correlations of the functional lateralization of the IPS (**B**) and cerebellum (**C**) with reading speed. IPS = inferior parietal sulcus, ^+^ FDR corrected *p* < 0.1, * FDR corrected *p* < 0.05, ** FDR corrected *p* < 0.01.

The raw scores on reading skills were converted into standard Z scores for subsequent analysis. Partial correlation analyses indicated that activation of the anterior Exner’s area during writing (*r* = −0.55, *p* = 0.070, power = 0.807) and drawing tasks (*r* = −0.71, *p* = 0.002, power = 0.983) was correlated with character recognition scores after controlling for age and IQ (Figure 4A). Partial correlation analyses indicated that activation of the anterior Exner’s area during handwriting (*r* = −0.55, *p* = 0.070, power = 0.807) and drawing tasks (*r* = −0.71, *p* = 0.002, power = 0.983) was correlated with character recognition after controlling for age and IQ (Figure 4A).

In addition to regional activation, we found that functional lateralization of the IPS during handwriting (*r* = −0.57, *p* = 0.021, power = 0.648) but not during drawing (*r* = −0.47, *p* = 0.186, power = 0.844) was negatively correlated with reading speed (Figure 4B). Similarly, the functional lateralization of the cerebellum during handwriting (*r* = 0.58, *p* = 0.021, power = 0.864) but not during drawing (*r* = 0.21, *p* = 0.913, power = 0.16) was positively correlated with reading speed (Figure 4C).

## 4. Discussion

Using fMRI and a copying task, we aimed to elucidate brain correlates of Chinese handwriting in children and to determine the associations between the neural circuitry supporting handwriting and reading development. We found that Exner’s area, the primary motor area, the IPS, FG, and the cerebellum were activated during handwriting in children. Such an activation pattern is similar to that reported in adults, suggesting that the brain network involved in handwriting has been established by middle childhood. Moreover, the key brain regions responsible for handwriting exhibited a complex lateralization of activation in children, suggesting that functional lateralization has not yet been established. Interestingly, unlike adults, children failed to show specific brain activation and functional lateralization in motor and visual regions when writing relative to when drawing symbols, suggesting a lack of functional specialization for handwriting. Finally, through a series of brain-and-behavior correlation analyses, we found that brain activation in Exner’s area during handwriting and drawing tasks and functional lateralization of the IPS and cerebellum were associated with individual variation in reading skills, suggesting that the handwriting-related neural network is associated with reading processing in children. Overall, this study comprehensively characterized the neural correlates of Chinese handwriting in children and their relation to reading development, advancing our understanding of the neural basis of the development of handwriting and reading skills. 

### 4.1. Brain Activation during Chinese Handwriting in Children

Analysis of whole-brain activation revealed that compared to rest, multiple motor regions including the bilateral premotor area, primary motor area, posterior parietal lobule, inferior and middle occipital gyrus, FG, and cerebellum were significantly activated during handwriting and drawing. These brain regions are critical for adult handwriting in Chinese [32] and in alphabetic languages [7,14]. Accordingly, we concluded that the brain network supporting handwriting isestablished by middle childhood. Unexpectedly, we did not find differences in brain activation between handwriting and drawing tasks, even though a lenient statistical threshold was used. This result contradicts prior research in adults showing greater activation of motor and visual regions during handwriting than when drawing symbols [31,32], despite the overlap in brain networks supporting these skills [46]. The specific brain activation elicited by handwriting may be shaped by long-term practice in adults. However, the lack of differences in brain activation between handwriting and drawing in children in the present study suggests that handwriting-specific brain activation patterns are not established by middle childhood. Furthermore, we also did not observe differences in brain activation between the HFC and LFC conditions, inconsistent with our previous findings in adults that showed greater activation in the frontal motor cortices, superior parietal lobule, and FG in the LFC condition than in the HFC condition [47]. The frequency effect was found to occur at the orthographic output level during handwriting [48]. The lack of a brain activation frequency effect in children suggests that the orthographic representation of the entire character has not been specialized, and therefore, regional activation of the handwriting network in children is not sensitive to the variety of character frequencies when writing.

### 4.2. Brain Lateralization during Chinese Handwriting in Children

Functional lateralization is an essential principle of brain organization. Motor skills are dominated by activity in the left hemisphere in most right-handers [49]. Such leftward asymmetry is critical to the development of motor skills [50]. A prior study of adults indicated that handwriting involves left-lateralized activation of Exner’s area, the IPL/SPL, and the FG and right-lateralized activation of the posterior cerebellum. Furthermore, Exner’s area was found to show handwriting-specific left lateralization relative to drawing [9]. In accordance with the adult results, we found left lateralized activation of Exner’s area and the IPS during handwriting in children. Thus, we posit that the lateralization pattern of these regions has emerged by middle childhood. However, the lateralization of Exner’s area and the IPS is not fully established by this period, because the lateralization was not significantly above the criterion of 0.2. This view is in line with the finding that French speakers show significant differences in activation lateralization between children and adults [14]. 

On the other hand, we found that the cerebellum, middle frontal gyrus, and FG showed right lateralization of activation during handwriting and drawing. First, consistent with the results in adults [7,32], we found that the cerebellum exhibited significant right lateralization during handwriting and drawing in children, suggesting that functional lateralization of the cerebellum develops by middle childhood. The cerebellum has been suggested to be predominantly engaged in ipsilateral motor execution [51]; thus, the right-lateralized activation observed in our study, in which all the participants used their dominant (right) hand to executive motor responses, is reasonable. 

In addition, we found that the middle frontal gyrus showed right-lateralized activation during handwriting, which is different from the left-lateralized activation observed in alphabetic languages [7,9,52]. Moreover, the left MFG was activated to a greater extent during Chinese handwriting than during English handwriting [30], suggesting that this structure plays a language-specific role in Chinese handwriting. Functionally, the left middle frontal gyrus has been proposed to serve other linguistic functions, such as phonological-graphemic conversion [7] and long-term orthographic representation [10]. However, we observed right-lateralized activation of the MFG in children, in contrast to the left-lateralized activation observed in adults. We tentatively interpret this discrepancy as stemming from the undeveloped advantage of the left MFG during handwriting in children. Consistent with our view, a recent study reported that right-lateralized activation in language networks in children decreased with age [53].

Ultimately, we found right-lateralized activation of the FG in children, which is consistent with the findings in adults [32]. However, this result contradicts findings of left lateralization of the FuG in alphabetic languages [7,9]. We conclude that the right-lateralized activation of the FuG may represent a language-specific neural marker of handwriting. Our finding extends previous findings of the difference in FG activation between Chinese and English speakers [30]. In Chinese, the right FG supports visual-orthographic processes during reading and writing [30,31]. A prior study also demonstrated that the right FG was involved in the integration of radicals into Chinese characters [54]. The segregation and integration of sub-character components during Chinese handwriting is demanding, leading to greater involvement of the right FG.

Our findings have implications for theories of neurodevelopment focusing on Chinese handwriting. Although we found adult-like activation patterns in children, activation lateralization in key regions related to handwriting was different from that reported in adults, suggesting that the automatization of Chinese handwriting requires a long period of practice. In addition, we revealed unique brain correlates of child handwriting in Chinese compared to alphabetic languages [14], providing new evidence for the language-specific hypothesis of the neural basis of language from the perspective of writing [55,56].

### 4.3. The Relationship between Neural Correlates of Handwriting and Reading Skills

In accordance with our hypothesis, we found that the regional activation of the anterior Exner’s area (−26, 2, 58) during handwriting and drawing was negatively correlated with character recognition, that is, individuals with higher reading skills exhibited lower activation of Exner’s area during handwriting and drawing tasks. This negative association can be explained by the inverse relationship between brain activity and motor automatization, namely, that stronger brain responses represent lower handwriting processing capacity. Alternatively, the negative association could suggest the functional dissociation of this region between reading and handwriting, despite the spatial overlap of brain activation. Consistent with this view, a recent study reported that handwriting practice may strengthen the development of neural specification for reading through segregation [57]. Exner’s area is a vital region for the writing–reading connection asit is activated during both reading and writing processing [21,30]. Furthermore, a transcranial magnetic stimulation (TMS) study reported that the disruption of Exner’s area affected the visual recognition of pseudowords, confirming the causal role of this region in reading processing [58]. This region is considered as the neural substrate of the motor representation of written scripts, which is automatically activated to facilitate visual recognition [21,24]. Moreover, Exner’s area may underlie the serial cognitive process shared by handwriting and reading [58]. Further studies are needed to specify the roles of Exner’s area in terms of the writing–reading connection. 

A novel finding of this study is that functional lateralization of the IPL and cerebellum during handwriting (but not drawing) was correlated with reading abilities in children, suggesting a specific neural mechanism underlying the connection between handwriting and reading. Functional lateralization is a pivotal neural signature of functional development that is thought to facilitate the efficient processing of information [59]. However, we observed a negative correlation between the degree of leftward asymmetry of the IPS during handwriting and reading skills. The negative correlation may have arisen because the IPS underpins the complex visuospatial analysis required during handwriting and reading, which is dominated by the right hemisphere [60,61]. 

Moreover, the rightward asymmetry of visuospatial skills increases with development during childhood and adolescence [61]. We found that the degree of left lateralization of cerebellar activation was positively related to reading skills, suggesting that greater involvement of the left cerebellum led to more proficient reading processing. Although previous studies have indicated the role of right-lateralized activation of the cerebellum in language development [62,63], dysfunction of the left cerebellum has been evidenced during reading [64] and motor tasks [65] in Chinese individuals with dyslexia, suggesting a potential compensatory role of the left cerebellum in Chinese reading [63]. Consequently, the right-lateralized activation of the cerebellum during handwriting may contribute to reading development by strengthening the shared functional lateralization of the IPS and the cerebellum. Further studies are needed to explore the specific mechanisms by which functional lateralization during handwriting impacts reading development.

In contrast to our hypothesis, the correlation of regional activation and lateralization of the FG with reading skills failed to reach statistical significance. This result suggests that the functional mechanism of visual-orthographic regions during handwriting may differ from the functions required during reading, despite spatial overlap in activation [12]. One possibility is that the shared activation of this region in handwriting and reading may only reflect the coactivation of this region in the two cognitive processes. This view is line with the dissociated hypothesis of dual orthographic representation for reading and writing [66]. Alternatively, the absence of a significant correlation may have been due to the small sample size and limited age range of participants. 

The development of reading proficiency involves complex interactions between multiple cognitive components. Due to the features of the language, Chinese reading necessitates unique neuroanatomical substrates [16]. Although numerous studies have suggested the potential contributions of handwriting to the development of Chinese reading [22,29], the underlying mechanisms by which handwriting facilitates reading development remain unclear. The establishment of shared neural correlates of handwriting and reading in children significantly extends the theoretical models of Chinese reading development. Moreover, our findings bolster the scientific foundation of using handwriting to treat dyslexia in Chinese students.

## 5. Limitations

Several limitations of this study should be taken into account when interpreting the current findings. First, the sample size was relatively small, which significantly limited the statistical power necessary to detect the correlation between brain activity during handwriting and reading. Further validation in a large sample of participants is needed. Second, Chinese is a logographic language that significantly differs in visuospatial attributes of written scripts and depth of orthography. Thus, the applicability of the identified correlation between handwriting-related brain activation and reading skills in Chinese to alphabetic languages remains unclear. Future studies are needed to evaluate the writing–reading connection.

## 6. Conclusions

In the present study, we examined brain activation patterns during handwriting in children and how the neural circuitry supporting handwriting is related to reading in Chinese speakers. We found that children in middle childhood exhibited adult-like patterns of brain activation during handwriting. However, activation lateralization of the key brain regions for handwriting differed from that reported in adults, suggesting that functional lateralization is still developing in middle childhood. Interestingly, we found that children did not exhibit differences in brain activation and lateralization during handwriting compared to drawing, suggesting a lack of functional specialization of the handwriting network in children. Finally, we identified the neural basis of the connection between handwriting and reading in Chinese. Overall, these findings provide new insight into the neural mechanisms of handwriting and their relation to reading.

## Figures and Tables

**Table 1:** Demographic information and behavioral task performance.

Variable	Mean (Standard Deviation)
Age (years)	10.40 (0.54)
Sex (male/female)	15/21
IQ	111.81 (15.56)
Handwriting skills	
Writing quality	25.55 (6.08)
Copying speed (in s)	101.49 (27.18)
Writing fluency (sentences)	27.64 (4.82)
Writing fluency (digits)	58.50 (12.20)
Reading skills	
Character recognition	2908.46 (261.60)
Reading speed (characters)	100.58 (19.57)

## Data Availability

The data that support the findings of this study are available from the corresponding author upon reasonable request.
